# Genomic Evidence of In-Flight Transmission of SARS-CoV-2 Despite Predeparture Testing

**DOI:** 10.3201/eid2703.204714

**Published:** 2021-03

**Authors:** Tara Swadi, Jemma L. Geoghegan, Tom Devine, Caroline McElnay, Jillian Sherwood, Phil Shoemack, Xiaoyun Ren, Matt Storey, Sarah Jefferies, Erasmus Smit, James Hadfield, Aoife Kenny, Lauren Jelley, Andrew Sporle, Andrea McNeill, G. Edwin Reynolds, Kip Mouldey, Lindsay Lowe, Gerard Sonder, Alexei J. Drummond, Sue Huang, David Welch, Edward C. Holmes, Nigel French, Colin R. Simpson, Joep de Ligt

**Affiliations:** New Zealand Ministry of Health, Wellington, New Zealand (T. Swadi, T. Devine, A. Kenny);; University of Otago, Dunedin, New Zealand (J.L. Geoghegan);; Institute of Environmental Science and Research, Porirua, New Zealand (J.L. Geoghegan, J. Sherwood, X. Ren, M. Storey, S. Jefferies, E. Smit, L. Jelley, A. McNeill, G. Sonder, S. Huang, J. de Ligt);; New Zealand Ministry of Health, Wellington (C. McElnay);; Bay of Plenty District Health Board, Tauranga, New Zealand (P. Shoemack, K. Mouldey, L. Lowe);; Fred Hutchinson Cancer Research Centre, Seattle, Washington, USA (J. Hadfield);; University of Auckland, Auckland, New Zealand (A. Sporle, A.J. Drummond, D. Welch);; iNZight Analytics Ltd., Auckland (A. Sporle);; Auckland District Health Board, Auckland (G.E. Reynolds);; The University of Sydney, Sydney, New South Wales, Australia (E.C. Holmes);; Massey University, Palmerston North, New Zealand (N. French);; Victoria University of Wellington, Wellington (C.R. Simpson);; University of Edinburgh, Edinburgh, UK (C.R. Simpson)

**Keywords:** 2019 novel coronavirus disease, coronavirus disease, COVID-19, severe acute respiratory syndrome coronavirus 2, SARS-CoV-2, viruses, respiratory infections, zoonoses, in-flight transmission, New Zealand

## Abstract

Since the first wave of coronavirus disease in March 2020, citizens and permanent residents returning to New Zealand have been required to undergo managed isolation and quarantine (MIQ) for 14 days and mandatory testing for severe acute respiratory syndrome coronavirus 2 (SARS-CoV-2). As of October 20, 2020, of 62,698 arrivals, testing of persons in MIQ had identified 215 cases of SARS-CoV-2 infection. Among 86 passengers on a flight from Dubai, United Arab Emirates, that arrived in New Zealand on September 29, test results were positive for 7 persons in MIQ. These passengers originated from 5 different countries before a layover in Dubai; 5 had negative predeparture SARS-CoV-2 test results. To assess possible points of infection, we analyzed information about their journeys, disease progression, and virus genomic data. All 7 SARS-CoV-2 genomes were genetically identical, except for a single mutation in 1 sample. Despite predeparture testing, multiple instances of in-flight SARS-CoV-2 transmission are likely.

In response to the growing international risks associated with importation of coronavirus disease (COVID-19), on March 20, 2020, New Zealand closed its borders to all but New Zealand citizens, permanent residents, and persons with an exemption (*1*). On April 9, 2020, to better control importation risks, New Zealand implemented a system of managed isolation and quarantine (MIQ) at the border. Persons arriving in New Zealand were required to stay in a government-assigned MIQ facility for at least 14 days before entering the New Zealand community. In June 2020, a system of testing persons who were returning to New Zealand and staying in MIQ facilities was instituted; nasopharyngeal swabs were taken on approximately the third and the twelfth day of the quarantine period and from anyone in whom symptoms developed or those identified as close contacts of persons with severe acute respiratory syndrome coronavirus 2 (SARS-CoV-2) positive test results.

 On September 29, 2020, flight EK448, which originated in Dubai, United Arab Emirates, with a stop in Kuala Lumpur, Malaysia, landed in Auckland, New Zealand. During the required 14-day MIQ period, 7 passengers who had traveled on the flight received positive SARS-CoV-2 test results. The 7 passengers had begun their journeys from 5 different countries before a layover in Dubai; predeparture SARS-CoV-2 test results were negative for 5 (Figure 1). These 7 passengers had been seated within 4 rows of each other during the ≈18-hour flight from Dubai to Auckland. Because recent studies have reported conflicting findings of the risks associated with in-flight transmission (*2*–*4*), we undertook a comprehensive investigation to determine the potential source of infection of these travelers.

**Figure 1:**
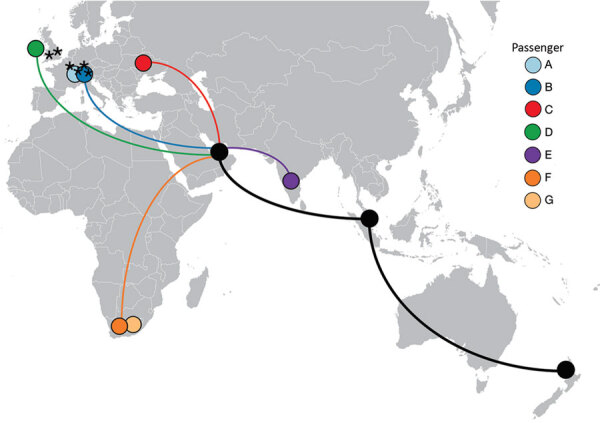
Countries of travel origins for 7 passengers who tested positive for severe acute respiratory syndrome coronavirus 2 infection after traveling on the same flight (EK448) from Dubai, United Arab Emirates, to Auckland, New Zealand, with a refueling stop in Kuala Lumpur, Malaysia, on September 29, 2020. Asterisks indicate where 6 other genetically identical genomes have been reported (*5*).

## Methods

### Case Details and Consent

In New Zealand, COVID-19 is a notifiable disease; all positive cases are reported to the national surveillance system, enabling further public health investigation. All persons with COVID-19 described in this article were contacted, and they provided written or verbal consent for their data to be used in this article. Case data were collected under the Ministry of Health contract for epidemic surveillance. The 7 persons with COVID-19 are denoted here as passengers A–G (Tables 1, 2).

**Table 1:** Detailed information for 7 passengers with SARS-CoV-2 infection detected after being on flight EK448, Dubai, United Arab Emirates, to Auckland, New Zealand, September 29, 2020*

Variable	Passenger							
	A	B	C	D	E	F	G	
Genome	Identical	Identical	Identical†	1 additional mutation	Identical	Identical	Identical	
Genome ID (GISAID accession no.) (*5*)	20CV0408 (EPI_ISL_ 582019)	20CV0409 (EPI_ISL_ 582020)	20CV0410 (EPI_ISL_ 582021)	20CV0401 (EPI_ISL_ 582018)	20CV0398 (EPI_ISL_ 582017)	20CV0414 (EPI_ISL_ 582022)	20CV0415 (EPI_ISL_ 582023)	
Preflight testing result (date)‡	Negative (Sep 24)	Negative (Sep 24)	Negative (Sep 25)	Negative (Sep 24)	Not tested	Negative (Sep 25)	Not tested	
Symptom onset date	Oct 1	Oct 2	Asymptomatic	Oct 4	Asymptomatic	Oct 3	Oct 9	
Date tested positive	Oct 2	Oct 2	Oct 2	Oct 7	Oct 6	Oct 8	Oct 8	
Technology§ and C_t_	GeneXpert, E-gene C_t_ 14.3, N2-gene C_t_ 16.4	GeneXpert, E-gene C_t_ 27, N2-gene C_t_ 29.3	GeneXpert, E-gene C_t_ 33.3, N2-gene C_t_ 36.8	GeneXpert, E-gene C_t_ 18.5 N2-gene C_t_ 20.4	GeneXpert, E-gene C_t_ 18.5, N2 gene C_t_ 22.3	BD Max, N1-gene C_t_ 22.0 N2-gene C_t_ 22.3	BD Max, N1-gene C_t_ 22.1, N2-gene C_t_ 19.1	
Country of origin	Switzerland	Switzerland	Ukraine	Ireland	India	South Africa		South Africa
Layover time in Dubai	9 h 27 min	9 h 27 min	11 h 30 min	8 h 18 min	70 h 54 min	5 h 44 min		5 h 44 min
Seat no. on flight	26G	26D	24C	27D	28G	24D/E/F/G		
PPE worn on airplane and bus‡	Face mask and gloves¶	Face mask and gloves¶	Not reported	Face mask and gloves	Not reported	Face mask		Face mask
Bus from airport to MIQ#	Bus 1	Bus 1	Bus 1 briefly, transported on bus 2	Bus 1	Bus 3	Bus 2		Bus 2

*GISAID, https://www.gisaid.org. C_t_, cycle threshold; MIQ, managed isolation and quarantine; PPE, personal protective equipment.  †Partial genome obtained (1 amplicon failed, resulting in 1,200 ambiguous nucleotide bases) but has the 5 defining mutations of the cluster.  ‡Self-reported.  §GeneXpert, https://www.cepheid.com; BD Max, https://www.bd.com.  ¶Reportedly removed when sleeping and seated.  #Social distancing and mandated mask wearing on all buses.

**Table 2:** Travel times for 7 passengers with SARS-CoV-2 infection detected after being on flight EK448, Dubai, United Arab Emirates, to Auckland, New Zealand, September 29, 2020

Variable	Date and time of departure country	Date and time of New Zealand arrival*
Flight EK448	Departed Dubai Sep 28, 08:29 am	Departed Dubai, Sep 28, 5:29 pm
	Arrived Kuala Lumpur, Malaysia, Sep 28, 7:11 pm	Arrived Kuala Lumpur, Sep 29,12:11 am
	Departed Kuala Lumpur Sep 28, 9:03 pm	Departed Kuala Lumpur, Sep 29, 2:03 am
	Arrived Auckland, Sep 29, at 11:31 am	Arrived Auckland, Sep 29, 11:31 am
Passengers A and B	Depart Zurich, Switzerland, Sep 27, 3:25 pm	Departed Zurich Sep 28, 2:25 am
	Arrive Dubai, Sep 27, 11:02 pm	Arrived Dubai, Sep 28, 8:02 am
Passenger C	Departed Kiev, Ukraine, Sep 27, 3:16 pm†	Departed Kiev Sep 28, 1:16 am†
	Arrived Dubai, Sep 27, 8:59 pm	Arrived Dubai, Sep 28, 5:59 am
Passenger D	Departed Dublin, Ireland, Sep 27, 2:10 pm‡	Departed Dublin Sep 28, 2:10 am‡
	Arrived Dubai Sep 28, 12:05 am	Arrived Dubai Sep 28, 9:05 am
Passenger E	Departed Kochi, India, Sep 25, 8:21 am§	Departed Kochi Sep 25, 2:51 pm§ Arrived Dubai Sep 25, 6:35 pm
	Arrived Dubai, Sep 25, 10:35 am	Arrived Dubai, Sep 25, 10:35 am
Passengers F and G	Departed Johannesburg, South Africa, Sep 27, 5:10 pm¶	Departed Johannesburg Sep 28, 4:10 am¶
	Arrived Dubai Sep 28, 02:45 am	Arrived Dubai Sep 28, 11:45 am

*Daylight savings time zone (Greenwich mean time +13 hours). †Flight EK2354. ‡Flight EK162. §Flight 6E67. ¶Flight EK762, seats 29 D, E, F, and G.

### Clinical Data and Sample Collection

Case details were sourced from the national notifiable diseases database, EpiSurv (https://surv.esr.cri.nz/episurv/index.php). While in MIQ, all 86 passengers on the flight underwent real-time reverse transcription PCR (rRT-PCR) diagnostic testing for SARS-CoV-2 on day 3 and again on day 12 if the previous test result was negative. Cabin crew members departed New Zealand soon after their arrival and were therefore not tested. Investigations used information from rRT-PCR testing by using the Cepheid GeneXpert system (https://www.cepheid.com) and BD Max (https://www.bd.com). We determined seating plans by consulting the flight manifest for the Boeing 777–300ER aircraft and confirmed them by administering a questionnaire to passengers, asking where they actually sat.

### Genome Sequencing

Independent viral extracts were prepared by the Institute of Environmental Science and Research (Porirua, New Zealand) from the 7 positive respiratory tract samples in which SARS-CoV-2 was initially detected by rRT-PCR. We extracted RNA from SARS-CoV-2–positive samples and subjected it to whole-genome sequencing by following the 1,200-bp amplicon protocol (*6*) and Oxford Nanopore Rapid barcoding R9.0 sequencing (*7*). Genomic data are available on GISAID (*5*) (Table 1).

### Phylogenetic Analysis of SARS-CoV-2 Genomes

The lineage of the genomes obtained from the 7 passengers was determined by using pangolin version 2.0.8 (https://pangolin.cog-uk.io) and compared with genomes from the same lineage available on GISAID (*5*). Genomes were aligned by using MAFFT version 7 (*8*) and using the FFT-NS-2 progressive alignment algorithm. We estimated a maximum-likelihood phylogenetic tree by using IQ-TREE version 1.6.8 (*9*) and the Hasegawa-Kishino-Yano nucleotide substitution model (*10*) with a gamma distributed rate variation among sites (HKY+Γ), the best-fit model as determined by ModelFinder (*11*), and branch support assessment by using the ultrafast bootstrap method (*12*).

### Analysis of Disease Transmission Data

All times and dates reported here were converted to New Zealand daylight savings time (Greenwich mean time + 13 hours) (Table 2). The mean incubation period, defined as the duration between estimated dates of infection and reported symptom onset, has been reported as 5–6 days (range 1–14 days) (*13*). We assumed a 5-day incubation period for passengers A, B, D, E, F, and G, and a 3-day incubation period for passenger C. We considered the median presymptomatic infectious period to be <1–4 days unless a negative PCR result indicated otherwise (*14*).

## Results

### The Flight

Flight EK448 from Dubai, UAE to Auckland, New Zealand, was an 18-hour, 2-minute flight on a Boeing 777–300ER aircraft. It departed Dubai on September 28, 2020, at 5:29 pm; arrived in Kuala Lumpur on September 29 at 12:11 am to refuel; and departed Kuala Lumpur on September 29 at 2:03 am. No passengers entered or exited the aircraft during the 2-hour refueling period in Kuala Lumpur. The flight arrived in Auckland on September 29 at 11:31 am. During the flight and before departure in Dubai airport, mask use was not mandatory; passengers A, B, D, F, and G self-reported mask and glove use while on the airplane but passengers C and E did not. In the days before the flight, these 7 passengers (other than the 2 travel groups, 1 of which comprised passengers A and B and the other passengers F and G) had been in different countries and did not have any form of contact (Figure 1). Similarly, none of the passengers reported having been in close contact at the Dubai airport. Passengers F and G were part of a family travel group of 4, all of whom reported having changed seats within their row during the flight.

All passengers, with the exception of passenger E, were transferred by bus to an MIQ facility in Rotorua, New Zealand. All passengers reported wearing masks during the bus journeys. Passengers A, B, and D were on bus 1; passengers F and G were on bus 2. Passenger C was initially seated on bus 1 but was transferred to bus 2 before transit. Both buses departed Auckland at 12:05 pm and arrived in Rotorua at 3:00 pm. Passenger E traveled on bus 3 to an MIQ facility in Auckland. Seating on all buses was physically distanced where possible, and mask use was mandated.

### Testing and Disease Progression

Five passengers reported having received negative test results before departure (Table 1). A negative test result was mandatory according to airline regulations for passenger C, who traveled from Ukraine.

**Figure 2:**
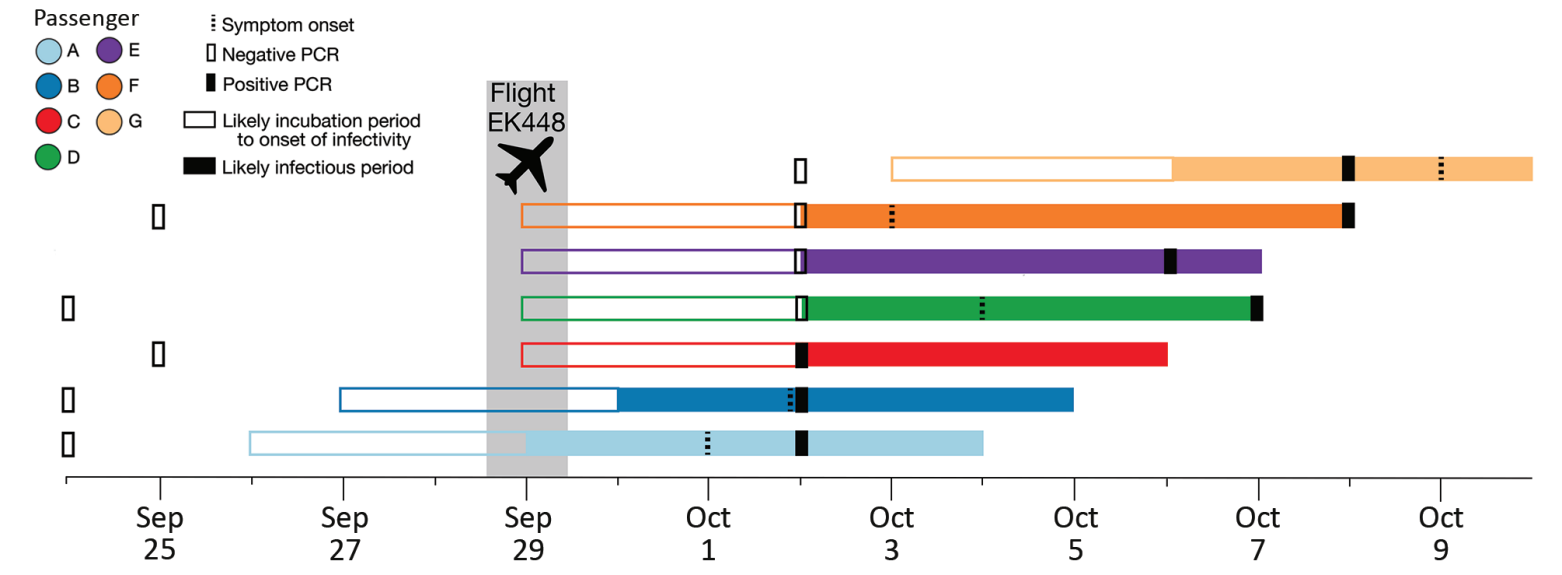
Timeline of likely incubation and infectious periods, indicating testing dates, for 7 passengers who tested positive for severe acute respiratory syndrome coronavirus 2 infection after traveling on the same flight (EK448) from Dubai, United Arab Emirates, to Auckland, New Zealand, with a refueling stop in Kuala Lumpur, Malaysia, on September 29, 2020.

The first 3 passengers to receive positive SARS-CoV-2 test results (passengers A, B, and C) were identified through routine surveillance testing on the third day of the quarantine period in New Zealand (Figure 2). Passengers A and B traveled together from Switzerland; both reported having had negative test results in their country of origin, <72 hours before boarding the flight. They departed Zurich, Switzerland, and arrived in Dubai on September 28, 2020, at 08:02 am. Passenger A reported symptom onset (general weakness and muscle pain) while in MIQ on October 1, and passenger B reported symptom onset (rhinorrhea, general weakness, cough, and muscle pain) on October 2. Test results for samples collected on October 2 from both persons were positive.

Test results for passenger C were also positive on October 2, but the passenger did not report symptoms at any time during the infection. This person had traveled from Kiev, Ukraine, and arrived in Dubai on September 28 at 5:59 am.

Test results for passenger D were negative on October 2, but the passenger reported symptoms on the fifth day after arrival in New Zealand. The symptoms progressively worsened, and another test on October 7 returned a positive result. Reported symptoms included coryza, headache, muscle pain, general weakness, irritability, confusion, and a head cold. This passenger had departed from Dublin, Ireland, and arrived in Dubai on September 28 at 9:05 am.

Test results for passenger E were negative on October 2, but the passenger was retested on October 6 as a potential close contact of those on the airplane and found to be positive for SARS-CoV-2. This passenger was not in the same MIQ facility (nor the same city) in New Zealand as the other passengers with reported cases and did not report symptoms during the infection. This passenger had departed from Kochi, India, and arrived in Dubai on September 25 at 6:35 pm.

Test results for passengers F and G (part of a group of 4 family members traveling together) were negative on October 2 in New Zealand. Passenger F became mildly symptomatic (coryza and a cough) on October 2 and self-reported having had a negative test result before leaving South Africa. The group was retested as potential contacts of those on the flight with positive results, and on October 8, results were positive for passengers F and G. Passenger G reported coryza and a sore throat on October 9. The 4-person travel group had departed from Johannesburg, South Africa, and arrived in Dubai on September 28 at 11:45 am. The 4 family members were seated in 4 adjacent seats in row 24 but interchanged seats within the row, such that no specific seat can be determined for each passenger (Figure 2). Test results were positive for only 2 of the 4 family members; after receiving the positive results, the persons were separated in the MIQ facility.

### Timeline of Transmission Events

**Figure 3:**
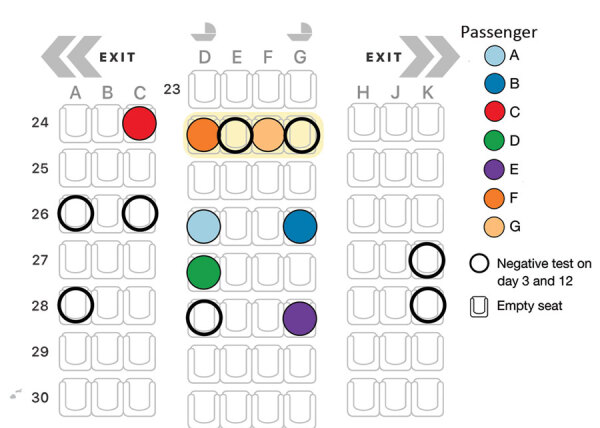
Seating arrangement (Boeing 777–300ER) for 7 passengers who tested positive for severe acute respiratory syndrome coronavirus 2 (SARS-CoV-2) infection on flight EK448 from Dubai, United Arab Emirates, to Auckland, New Zealand, with a refueling stop in Kuala Lumpur, Malaysia, on September 29, 2020. Passengers F and G interchanged seats within row 24. Open circles represent nearby passengers who were negative for SARS-CoV-2 on days 3 and 12 while in managed isolation and quarantine. All other seats shown remained empty.

The first person to experience symptoms was passenger A on October 1, consistent with having been infectious while on flight EK448 2 days earlier (Figure 3). The second person to experience symptoms, on October 2, was passenger B, a travel companion of passenger A, which may represent shared exposure to a source A, such that passenger B’s infection is not considered a case of in-flight transmission. Passenger C was asymptomatic and received a positive test result on day 3. Symptom onset and positive test result dates for passengers D, E, and F were all consistent with in-flight transmission. Passenger G was a travel companion of passenger F, and their date of symptom onset was consistent with infection during their stay in an MIQ facility, where they resided in the same room. As such, passenger G’s infection was not considered a result of in-flight transmission.

### Viral Genomic Data

**Figure 4:**
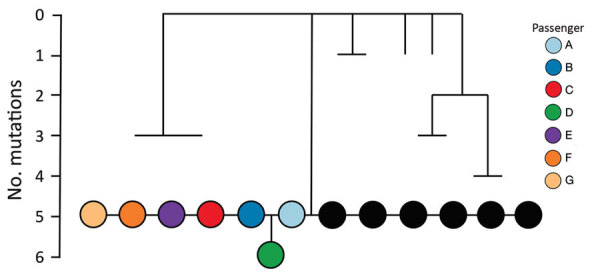
Simplified maximum-likelihood phylogenetic tree of genomes from severe acute respiratory syndrome coronavirus 2 from 7 passengers who traveled on flight EK448 (Boeing 777–300ER) from Dubai, United Arab Emirates, to Auckland, New Zealand, with a refueling stop in Kuala Lumpur, Malaysia, on September 29, 2020. Tree shows positive cases along with their closest genomic relatives sampled from the global dataset. Black circles illustrate cases obtained from the global dataset that are genetically identical, sampled September 2–23, 2020. Scale bar shows the number of mutations relative to the closest reconstructed ancestor from available global data.

**Figure 5:**
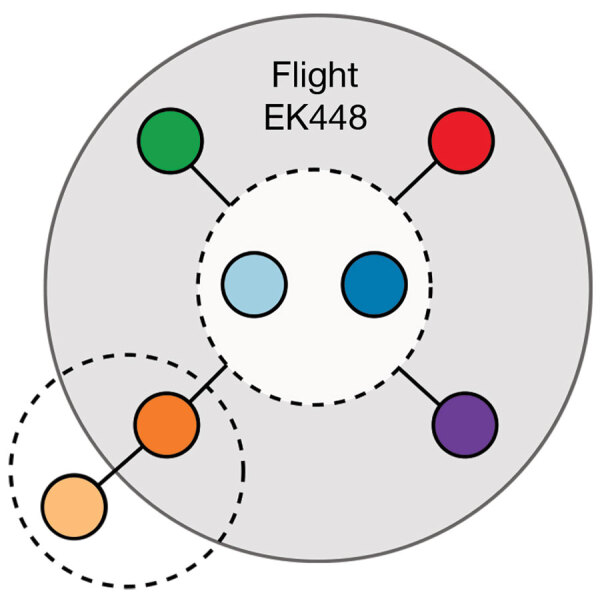
Network of likely severe acute respiratory syndrome coronavirus 2 (SARS-CoV-2) transmission among 7 passengers who traveled on flight EK448 (Boeing 777–300ER) from Dubai, United Arab Emirates, to Auckland, New Zealand, with a refueling stop in Kuala Lumpur, Malaysia, on September 29, 2020. The gray shaded area illustrates likely in-flight virus transmission. Dashed circles represent likely virus transmission between travel companions.

All SARS-CoV-2 samples from the 7 passengers were subjected to whole-genome sequencing for surveillance purposes. The sequences obtained were assigned to lineage B.1 and were genetically identical, apart from 1 mutation for the sample from passenger D (Figure 4) (*15*). By comparing these 7 genomes to the international database (GISAID), we identified 6 additional identical genomes: 4 from Switzerland and 2 from the United Kingdom, sampled during September 2–23. These findings were consistent with virus introduction onto the airplane from Switzerland by passenger A, B, or both (Figure 5). Nevertheless, accurately identifying the source of this outbreak may be impeded by substantial biases and gaps in global sequencing data (J. Geoghegan, unpub. data, https://www.medrxiv.org/content/10.1101/2020.10.28.20221853v1); hence, we cannot explicitly exclude passenger C as the source.

## Discussion

Evidence of in-flight transmission on a flight from the United Arab Emirates to New Zealand is strongly supported by the epidemiologic data, in-flight seating plan, symptom onset dates, and genomic data for this group of travelers who tested positive for SARS-CoV-2 (passengers A–G). Among the 7 passengers, 2 (A and B) were probably index case-patients infected before the flight, 4 (C, D, E, and F) were probably infected during the flight, and the remaining passenger (G) was probably infected while in MIQ. All 7 passengers were seated in aisle seats within 2 rows of where the presumed index case-patient(s) were seated.

Combined, these data present a likely scenario of >4 SARS-CoV-2 transmission events during a long-haul flight from Dubai to Auckland. These transmission events occurred despite reported in-flight use of masks and gloves. Further transmission between travel companions then occurred after the flight, in an MIQ facility.

These conclusions are supported by genome sequencing, an in-flight seating plan, and dates of disease onset. These data do not definitively exclude an alternative exposure event, such as virus transmission at the Dubai airport before boarding (e.g., during check-in or in boarding queues). However, the close proximity of the relevant passengers on board suggests that in-flight transmission is plausible.

Similar reports of SARS-CoV-2 being transmitted during flight have recently been published (*3*,*4*,*16*,*17*). Those reports, along with the findings we report, demonstrate the potential for SARS-CoV-2 to spread on long-haul flights. It must also be noted that the auxiliary power unit of the flight EK448 aircraft was reported as having been inoperative for ≈30 minutes during the 2-hour refueling stop in Kuala Lumpur, such that the environmental control system would not have been working during this period.

That 3 passengers had positive test results on day 3 of their 14-day quarantine period indicates some of the complexities of determining the value of predeparture testing, including the modality and timing of any such testing. Although not definitive, these findings underscore the value of considering all international passengers arriving in New Zealand as being potentially infected with SARS-CoV-2, even if predeparture testing was undertaken, social distancing and spacing were followed, and personal protective equipment was used in-flight.
